# Approaches to vaccination against *Theileria parva* and *Theileria annulata*

**DOI:** 10.1111/pim.12388

**Published:** 2016-12-21

**Authors:** V. Nene, W. I. Morrison

**Affiliations:** ^1^ The International Livestock Research InstituteNairobiKenya; ^2^ The Roslin InstituteRoyal (Dick) School of Veterinary StudiesUniversity of EdinburghMidlothianUK

**Keywords:** CD8 T cell, neutralizing antibody, *Theileria annulata*, *Theileria parva*, vaccination

## Abstract

Despite having different cell tropism, the pathogenesis and immunobiology of the diseases caused by *Theileria parva* and *Theileria annulata* are remarkably similar. Live vaccines have been available for both parasites for over 40 years, but although they provide strong protection, practical disadvantages have limited their widespread application. Efforts to develop alternative vaccines using defined parasite antigens have focused on the sporozoite and intracellular schizont stages of the parasites. Experimental vaccination studies using viral vectors expressing *T. parva* schizont antigens and *T. parva* and *T. annulata* sporozoite antigens incorporated in adjuvant have, in each case, demonstrated protection against parasite challenge in a proportion of vaccinated animals. Current work is investigating alternative antigen delivery systems in an attempt to improve the levels of protection. The genome architecture and protein‐coding capacity of *T. parva* and *T. annulata* are remarkably similar. The major sporozoite surface antigen in both species and most of the schizont antigens are encoded by orthologous genes. The former have been shown to induce species cross‐reactive neutralizing antibodies, and comparison of the schizont antigen orthologues has demonstrated that some of them display high levels of sequence conservation. Hence, advances in development of subunit vaccines against one parasite species are likely to be readily applicable to the other.

## 1 Introduction

*Theileria* are tickborne apicomplexan parasites found in tropical and subtropical regions of the world, where they predominantly infect ruminants.^1^, ^2^, ^3^ Wild and domestic ruminants harbour a large number of *Theileria* species, but only a few species, most notably *Theileria parva* and *Theileria annulata* in cattle and *Theileria lestoquardi* (previously also known as *Theileria hirci*) in sheep, are associated with severe clinical disease in farm animals.^4^, ^5^, ^6^, ^7^, ^8^ The diseases caused by these parasites are of major economic importance in the regions where they occur. Their geographical distribution is determined largely by that of the respective tick vector species. *Theileria parva*, which is transmitted predominantly by *Rhipicephalus appendiculatus* ticks, occurs in eastern and southern Africa, whereas *T. annulata*, transmitted by several species of *Hyalomma* ticks, occurs around the Mediterranean basin, north‐east Africa, the Middle East, India and southern Asia. *Theileria lestoquardi*, which is closely related to *T. annulata* and is transmitted by the same tick species,^9^, ^10^ appears to have a more restricted distribution, reports of infection being confined mainly to the Middle East and north‐east Africa. *Theileria parva* and *T. annulata* also infect the African buffalo (*Syncerus caffer*) and the Asian buffalo (*Bubalus bubalus*), respectively. The latter sometimes suffer mild clinical disease,^11^ whereas *T. parva* is nonpathogenic in the African buffalo, but infected buffalo represent an important wildlife reservoir for infection of cattle.^12^

The pathogenic *Theileria* species cause acute lymphoproliferative diseases, with high levels of morbidity and mortality in susceptible populations of animals.^6^, ^13^, ^14^ Like malaria parasites, *Theileria* undergo sequential development in nucleated cells and erythrocytes, but pathogenicity is largely attributable to parasite development during the nucleated cell stage. *Theileria* invade leucocytes, but unlike most other apicomplexan parasites, they reside free within the cytosol of the host cells.^15^ Development to the schizont stage induces activation and proliferation of the infected host leucocytes,^16^ and, by associating with the mitotic spindle during cell division, the parasites are able to divide at the same time as the host cells, ensuring that infection is retained in the daughter cells.^17^, ^18^, ^19^ This process facilitates rapid parasite multiplication prior to differentiation to the erythrocyte‐infective merozoite stage. In susceptible animals, large numbers of infected cells are found in the local lymph node draining the site of infection, from which they disseminate throughout the lymphoid system and to nonlymphoid tissues.^20^ Infection usually results in death within 3–4 weeks. The mode of replication of the schizont stage of *T. parva*,* T. annulata* and *T. lestoquardi* enables the parasitized cells of these species to be cultured in vitro as continuously growing cell lines.^21^ These Theileria are frequently referred to as “transforming species”. Other species such as *Theileria mutans* and *Theileria veliferi*, which rarely cause disease, multiply predominantly during the intra‐erythrocytic stage of development and undergo limited replication in nucleated cells (reviewed in Ref. 22).

The intra‐erythrocyte piroplasm stage of *T. parva* undergoes little or no multiplication, whereas there is some replication of *T. annulata* piroplasms,^23^ which is associated with higher levels of infection of erythrocytes. Infections with *T. annulata* may result in moderate anaemia and occasionally jaundice, although pathology produced by the schizont stage is usually the primary cause of mortality by both species.

Because of the acute and fatal nature of the *Theileria* infections in susceptible stock, control of the diseases is particularly challenging. In the past, prevention of tick infestation by application of acaricides has been used successfully to prevent disease. However, the need for almost continuous use of these chemicals has proved to be expensive and difficult to sustain and runs the risk of selecting acaricide‐resistant tick populations. A single therapeutic compound (buparvaquone, marketed as Buparvex) is available,^24^ but its use is limited by cost and the need to treat animals during the early stages of disease to be effective. Moreover, there are recent reports of the emergence of drug‐resistant strains of *T. annulata*.^25^, ^26^

Due to the shortcomings of these control measures, it has long been recognized that vaccination is the most sustainable option for control of these diseases. Live vaccines were produced for *T. parva* and *T. annulata* over 40 years ago,^5^, ^27^, ^28^ but have a number of practical disadvantages that have limited their use in many areas. Efforts to develop vaccines based on use of defined antigens have so far met with limited success. These recent studies have been the subject of several recent reviews.^29^, ^30^, ^31^ Herein, we will discuss the current status of vaccination against *T. parva* and *T. annulata* and consider the potential value of comparative studies of these parasites for future development of improved vaccines.

## 2 *Theileria parva* and *Theileria annulata* Infect Different Cell Types But Cause Similar Immunopathology

The ability to infect bovine cells in vitro with tick‐derived sporozoites has enabled the cell tropism of the different *Theileria* species to be determined. Early studies demonstrated that *T. parva* sporozoites can bind to and infect B and all subsets of T lymphocytes in vitro with similar efficiency, whereas *T. annulata* was found to infect monocytes and B lymphocytes but not T lymphocytes.^32^, ^33^ Subsequent analyses of the cells infected by *T. parva* in vivo showed that the vast majority of the infected cells in animals undergoing primary infection were T cells, of both CD4 and CD8 T‐cell lineage.^34^ Moreover, experiments in which purified cell populations were infected in vitro with sporozoites and then administered to the autologous animals after 24 hours (when no viable sporozoites remained) demonstrated that infected T cells produced lethal infections, whereas infected B cells resulted in transient mild infection from which they recovered.^35^ In the animals that received *T. parva*‐infected B cells, infection was first detected in the regional lymph node around the same time as in animals receiving infected T cells, but they were able to clear the infection around 11–12 days after infection and were subsequently immune to parasite challenge. We have recently shown that animals inoculated with infected autologous T cells that have been cultured in vitro for 6 weeks or more also develop similar mild self‐limiting infections (Morrison WI and Connelley T, unpublished data). Based on these findings, we have concluded that transformation of the infected cells alone is not sufficient for virulence and that additional as yet undefined properties of recently infected T cells enable them to produce disease. Recent studies have identified a Zambian isolate of *T. parva* that infects CD8 T cells but not CD4 or γ/δ T cells.^36^ Infection with this isolate results in disease, although the authors suggested that it is of lower virulence than other isolates. The relative contribution of infected monocytes and B cells to infections with *T. annulata* in vivo has not been determined.

Despite the different cell tropisms of *T. parva* and *T. annulata*, the pathology they produce is remarkably similar. In both infections, the parasitized cells migrate from the site of infection and become disseminated throughout the lymphoid system. In both cases, there is also evidence of an early powerful nonspecific T‐cell response in animals experimentally infected with a lethal dose of sporozoites, but this response appears to be ineffective in controlling the infection. In the case of *T. parva,* the regional lymph node was found to contain 15%–20% lymphoblasts at a time when there was <1% parasitized cells.^37^ A majority of these cells were CD8 T cells, including a phenotypically unusual subset of CD2^−^CD8^+^ cells; they did not show any cytotoxic activity against parasitized cells and also failed to respond to antigenic or mitogenic stimulation in vitro. Separate studies of animals infected with *T. annulata* revealed a broadly similar picture.^38^, ^39^, ^40^ These observations have not been investigated further, nor has there been any comparison of the responses to infections induced by needle and tick challenge. Nevertheless, currently available data indicate that the progressive nature of infections with the two parasite species is not due to a failure to stimulate an immune response but rather that the infection stimulates a dysregulated immune response that fails to differentiate to generate appropriate effector functions. This presumably reflects a strategy by the parasites to delay parasite clearance. What is remarkable is that infection of different cell types by the two *Theileria* species results in a very similar outcome. Further research using contemporary methodologies is required to understand the molecular basis of these immune responses, to better inform what is required for appropriate differentiation of a protective vs a nonprotective immune response.

## 3 Live Vaccines

### 3.1 Vaccination with parasitized cell lines

The advent in the 1960s of cell culture systems to propagate *Theileria*‐infected cells as continuously growing cell lines in vitro opened up an obvious opportunity to investigate the use of these cells for vaccination. However, the results obtained with *T. annulata* and *T. parva* were very different. Administration of 10^5^ or fewer *T. annulata*‐infected cells resulted in immunity to parasite challenge, although prolonged in vitro passage of the cell lines was required to avoid production of clinical disease by the vaccine.^5^, ^27^, ^41^ Initial studies in Israel in the early 1970s resulted in a national vaccine programme for *T. annulata* using cultured cells. Similar vaccines were subsequently developed in several other countries using cell lines derived from local parasite isolates. These vaccines are generally produced in batches that are cryopreserved in aliquots in liquid nitrogen and thawed immediately before use. A dose of approximately 1–5 × 10^6^ cells (equivalent to 1–5 mL of cultured cells) is used, which allows considerable leeway for cell loss during freezing and thawing of the vaccine.^5^

By contrast to *T. annulata*, similar studies with *T. parva* demonstrated that a dose of 10^8^ cultured cells was required to generate immunity in all animals.^42^ Similar to *T. annulata*, in vitro passage of the cells (>50 passages) was required to avoid production of disease by the inoculated cells. As 10^8^ cells represented >100 mL of culture, this method of vaccination was not considered economically viable.

Subsequent studies demonstrated that, for both parasite species, transfer of the parasites from the donor culture cells into cells of the recipient animals is required for successful induction of immunity and that this transfer occurs at much lower frequency with *T. parva* than with *T. annulata*.^43^, ^44^ This reflects a requirement of protective T‐cell responses to recognize parasite antigens in the context of the major histocompatibility complex (MHC) of the recipient animal. Analyses of immune responses in vaccinated animals demonstrated that animals generated a CD8 T‐cell response directed against alloantigens on the inoculated cells at 7–10 days, followed by a second CD8 T‐cell response about a week later, which was parasite specific and restricted by the MHC of the recipient animal. The mechanisms by which the schizonts transfer from one cell to another are not known, nor is it clear why this occurs with greater efficiency in *T. annulata*.

### 3.2 Vaccination by infection with sporozoites

The unfeasibility of using cultured parasites for vaccination against *T. parva* prompted alternative methods of immunization with live parasites. With the development of methods to cryopreserve large stocks of sporozoites, in the form of homogenized infected ticks,^45^ titrations of this material in cattle were undertaken with the aim of identifying a dose that would reproducibly result in mild transient infections and immunity. However, this was not achievable because the lowest doses that produced infection in all animals still resulted in severe reactions in some of the animals.^46^ An alternative approach involving infection and simultaneous treatment with oxytetracycline was developed, which successfully achieved mild transient infections in all animals.^28^, ^47^ Use of a long‐acting formulation of oxytetracycline that provided 5–6 days of activity was required to control the infection. This so‐called infection and treatment immunization procedure resulted in long‐lasting immunity in all animals against high challenge doses of the *T. parva* isolate used for immunization, but only a proportion of immunized animals withstood challenge with other parasite isolates.^48^, ^49^ However, based on a series of experiments involving immunization and challenge of cattle with different combinations of parasite isolates, a mixture of three isolates was identified (known as the Muguga cocktail), which when used to immunize cattle gave broad protection against experimental challenge with different parasite isolates and against field challenge with *T. parva*. Despite evidence of efficacy,^50^ until recently use of the Muguga cocktail vaccine in the field has been limited.

### 3.3 Shortcomings of live *Theileria* vaccines

#### 3.3.1 Practical constraints

Vaccination using the Muguga cocktail requires production of three large batches of *T. parva* sporozoites by feeding ticks on cattle infected with each parasite isolate, and each batch needs to be carefully titrated in cattle to determine a dose that will reproducibly infect and immunize all animals but will not break through the tetracycline treatment. This complex protocol coupled with the requirement for a liquid nitrogen cold chain to distribute the vaccine presents challenges for quality control and marketing. However, recent initiatives have led to increased field uptake. This has included the establishment of a centre for vaccine production and systems to facilitate distribution of the vaccine.

#### 3.3.2 Parasite strain‐restricted immunity

As referred to above, vaccination against *T. parva* by infection and treatment was found to require incorporation of three parasite isolates in the vaccine to provide immunity against field challenge. This followed on from field studies in which animals immunized with a single parasite isolate were not protected, providing the first convincing evidence of antigenic heterogeneity in *T. parva*.^51^ More extensive testing of the Muguga cocktail vaccine has demonstrated that it does not provide complete protection against field challenge in all circumstances, in particular against challenge with buffalo‐derived parasites.^52^, ^53^ Indeed in one study, vaccinated animals introduced into an area grazed only by buffalo showed no protection.^52^ These findings are consistent with sequencing data on parasite genes encoding two polymorphic antigens (Tp1 and Tp2) and genomewide SNP density, which revealed much greater genotypic diversity in parasites isolated from buffalo compared with those of cattle origin.^54^

The results of two recent studies of the Muguga cocktail vaccine, one involving genomic sequencing of the three component parasites^55^ and the other based on high‐throughput sequencing of PCR amplicons of six genes encoding *T. parva* antigens (including Tp1 and Tp2),^56^ have indicated that the vaccine contains only a small component of the genetic and antigenic diversity detected in field populations of *T. parva*. Each of the three parasite isolates in the Muguga cocktail exhibited very limited diversity, and two of them (Muguga and Serengeti) showed a remarkably high level of sequence similarity, but differed significantly from the third isolate (Kiambu). The Serengeti parasite was originally isolated from a buffalo and adapted to tick transmission between cattle following several tick passages. However, the results of these two studies both suggested that the Serengeti isolate had at some point become contaminated with parasites from the Muguga isolate. Amplicon sequencing and satellite DNA typing also demonstrated that the vaccine components contained minor genotypic components present at <5% within the vaccine parasites.^56^ If these minor components contribute to the broad protective capacity of the vaccine, then the possibility that these components might not be present in all vaccine batches or indeed all vaccine doses is of concern regarding standardization of vaccine content. The authors proposed formulating an alternative vaccine comprising a mixture of antigenically divergent parasite clones, to standardize the content of the vaccine and potentially enhance its ability to generate broadly cross‐reactive immunity. This material would have to be derived through tick passage, and the influence on parasite homogeneity through sexual recombination would need to be investigated.

It is of note that antigenic heterogeneity among isolates of *T. annulata* has also been documented in a number of early studies, which showed incomplete cross‐protection between some isolates, with a proportion of the animals succumbing to disease (reviewed in Ref. 4, 5). Moreover, initial cell line vaccine testing in Israel showed that vaccinated animals challenged with heterologous parasite isolates developed more severe clinical reactions than those receiving homologous challenge, although the animals survived. More recently, experiments involving immunization of cattle with cell lines infected with Tunisian *T. annulata* isolates have indicated that the passage history of the cell line can influence the level of protection against heterologous isolates^57^; cattle immunized with cell lines that had undergone prolonged passage suffered more severe reactions following challenge than those immunized with the same cell lines at an earlier level of passage. Analyses of these cell lines with isoenzymes and polymorphic genotypic markers indicated that prolonged passage resulted in reduced genetic diversity in the parasites within the cell lines, and these authors suggested that this loss in diversity may account for the reduced capacity to provide protection against challenge with heterologous parasite isolates. Nevertheless, the reasons why strain‐restricted immunity, as observed with *T. parva,* is not a major issue when vaccinating cattle against *T. annulata* are unclear.

#### 3.3.3 Acceptability of live vaccines

A key feature of infections with *Theileria* parasites is their ability to establish persistent infections in the face of immune responses that control the infection. In the case of *T. parva* and *T. annulata*, persistent infections (referred to as the carrier state) are usually not detectable microscopically but can be revealed by polymerase chain reaction (PCR) assays and can be transmitted by ticks.^58^ The live parasites used for vaccination often retain the ability to establish a carrier state, although some of the *T. annulata* cell line vaccines that have been subjected to prolonged in vitro passage are claimed to have lost this property. Nevertheless, there has been great reluctance to use live parasites originating in one country to vaccinate animals in other countries, because of perceived risks of introducing “foreign parasite strains” of different antigenic composition or virulence. Hence, for *T. annulata*, each country has tended to develop its own cell line vaccine from a local parasite isolate. In the case of *T. parva*, use of the Muguga cocktail vaccine has been slow to gain acceptance outside Kenya and Tanzania. In the longer term, this issue could potentially be addressed by genetically modifying the parasite such that the carrier status is no longer maintained.

## 4 Approaches to Development of Subunit Vaccines

Because of the limitations of available live vaccines, efforts have been made to develop alternative vaccines based on the use of defined antigens. This has necessitated studies to understand the immune responses to the parasites and their role in immune protection. Much of this work has focused on the sporozoite and schizont developmental stages.

### 4.1 Protective immune responses to schizont‐infected cells

#### 4.1.1 Immune protection

There is a large body of evidence indicating that immunity generated by infection with *T. parva* or *T. annulata* is mediated by cellular immune responses directed against schizont‐infected leucocytes. This information has been reviewed elsewhere^29^, ^30^ and will therefore only be summarized briefly here.

Because animals can be immunized by administration of schizont‐infected cell lines, immunity is clearly not dependent on exposure to preschizont stages of the parasite. Moreover, immunization with live parasites, either cell lines or sporozoites, does not prevent establishment of infection, but rather schizont‐infected cells are often detected transiently 8–10 days after parasite challenge of immunized animals, indicating that immunity operates against this stage of the parasite.

Analyses of the immune responses *ex vivo* following challenge of *T. parva*‐immunized animals have demonstrated MHC‐restricted cytotoxic CD8 T‐cell responses against autologous *T. parva*‐infected cells, coinciding with parasite clearance.^59^, ^60^ Two further observations provided evidence that these T cells are key mediators of immunity to *T. parva*. First, transfer of responding CD8 T cells (but not CD4 T cells), from immune to naïve identical twin calves, was found to confer protection against parasite challenge in the naïve recipients.^61^ Second, the CD8 T‐cell responses showed parasite strain specificity that varied between animals immunized with the same parasite isolate, and correlated closely with the observed immunity following challenge with a cloned heterologous parasite strain.^62^, ^63^ Further studies have demonstrated high frequencies of parasite‐specific CD8 T‐cell precursors in the memory T‐cell populations of immune animals,^63^ and the specificities of these T cells have been studied in detail using CD8 T‐cell lines and clones derived from the memory populations.^64^, ^65^ Analyses of T‐cell lines obtained from immune animals by stimulation with autologous parasitized cells have also identified strong parasite‐specific CD4 T‐cell responses, which recognize antigen presented on the surface of infected leucocytes.^66^, ^67^ Findings from in vitro experiments involving mixing of CD4 and CD8 T cells from immune or naïve animals have indicated that the parasite‐specific CD4 T cells may provide help for induction and recall of parasite‐specific CD8 T‐cell responses.^68^

While the role of T‐cell responses in immunity to *T. annulata* has not been examined in the same detail, available evidence indicates a very similar profile of responses, namely cytotoxic CD8 T‐cell responses detected *ex vivo* following immunization and challenge with the parasite and the presence of CD4 and CD8 T cells specific for autologous parasitized cells in T‐cell lines generated in vitro from immune animals.^69^, ^70^

#### 4.1.2 The antigens recognized on schizont‐infected cells

A series of studies using *T. parva*‐specific CD8 T‐cell lines to screen for recognition of cells transfected with parasite cDNAs has identified 10 antigens (Table 1) that are recognized by CD8 T cells from immune cattle.^56^, ^71^ The genes encoding these antigens are unrelated and are distributed across the genome. Some are predicted to be orthologues of other parasite and/or mammalian genes, whereas others have no detectable orthologues. A striking feature of the antigen screening results was that animals of different MHC genotypes tended to detect different *T. parva* antigens, and, within the animals studied, only one of the antigens (Tp2) was recognized by T cells from animals of several different MHC genotypes. Epitope screening, in most cases, also identified a single dominant epitope recognized by T cells restricted by a given MHC allele.^72^ However, animals of some MHC types do not recognize any of these antigens. The results of these studies collectively indicate that a large number of *T. parva* proteins are capable of eliciting CD8 T‐cell responses and that many of these target antigens have not yet been identified.

**Table 1:** Level of amino acid sequence conservation between orthologues of schizont vaccine antigens in *Theileria parva* and *Theileria annulata*

*Theileria parva*			*Theileria annulata*			Ta/Tp amino acid identity^c^
Antigen	Amino acids	Epitope sequence	Antigen^a^	Amino acids	Epitope sequence^b^	
Tp1	543	^214^VGYPKVKEEML_224_	Ta1	529	^214^YKYPNIKQEML_224_	52%
Tp2	175	^27^SHEELKKLGML_37_	Ta2	178	^28^KDEELDAMGML_38_	60%
		^40^DGFDRDALF_48_			^41^DLNKELLFQ_49_	
		^49^KSSHGMGKVGK_59_			^50^QTSHILTKVGK_60_	
		^96^FAQSLVCVL_104_			^97^FAASIHCVA_105_	
		^98^QSLVCVLMK_106_			^99^ASIHCVANK_107_	
		^138^KTSIPNPCKW_147_			^139^KESIPNPCDW_148_	
Tp3	265	Unknown	Ta3	265		77%
Tp4	579	^328^TGASIQTTL_336_	Ta4	520	^328^TGASIQTTL_336_	96%
Tp5	155	^87^SKADVIAKY_95_	**Ta5**	155	^87^SKADVIAKY_95_	98.1%
Tp6	277	Unknown	Ta6	277		98.5%
Tp7	721	^206^EFISFPISL_214_	Ta7	722	^206^EFISFPISL_214_	97.5%
Tp8	434	^373^CGAELNHFL_381_	Ta8	409	^352^CGAELNHYL_360_	89%
Tp9	335	^67^AKFPGMKKS_75_	**Ta9**	336	^64^SKFPKMRMG_72_	64%
Tp10	443	^419^NNPELIPVL_427_	Ta10	447	^423^NNPELIPVL_431_	92%

a Only Ta5 and Ta9 (bold) confirmed as CD8 T‐cell antigens in *T. annulata*.

b Divergent amino acid residues are highlighted in grey.

c The percentage of amino acid residues conserved between the *T. parva* and *T. annulata* orthologues, based on the reference genome sequences.

Detailed analyses of the responses to two of the antigens, Tp1 and Tp2, which are recognized on the A18 and A10 MHCI backgrounds, respectively, demonstrated that in each case, approximately 70% of the parasite‐specific CD8 T cells in MHC‐homozygous immune animals were specific for the respective antigen and that these animals did not recognize the other defined antigens.^65^ Subsequent work demonstrated that these antigens are highly polymorphic and that the respective T‐cell responses are often parasite strain‐restricted. Elsewhere, we have discussed in further detail how this observed focusing of the CD8 T‐cell response on highly dominant polymorphic T‐cell epitopes is a key determinant of parasite strain specificity of CD8 T‐cell response.^65^, ^73^ However, recent unpublished studies (H. Hemmink, W.I. Morrison, P. Toye, T. Sitt and W. Weir, manuscript in preparation) have shown that several of the other *T. parva* CD8 T‐cell antigens are highly conserved among field isolates of the parasite.

Similar large‐scale antigen screening has not been undertaken with T cells specific for the other pathogenic *Theileria* species. However, screening for recognition of the orthologues of the 10 *T. parva* antigens in *T. annulata* 74 and *T. lestoquardi* 75 has been conducted with a limited number of CD8 T‐cell lines from cattle and sheep, respectively. In each case, positive results were obtained with two antigens (Tp5 and Tp9 orthologues in *T. annulata* and Tp8 and Tp9 orthologues in *T. lestoquardi*). An additional *T. annulata* antigen was identified by screening a small number of parasite cDNAs. Analyses of sequences of Tp9 and the orthologue in *T. annulata* in different parasite isolates have shown that this antigen is highly polymorphic in both parasite species 56, 74 and that the corresponding CD8 T‐cell lines exhibit parasite strain specificity. Although the repertoire of antigens recognized by CD8 T cells specific for these parasites is far from complete, these initial findings suggest that there is likely to be substantial commonality in the gene products that they recognize. Hence, information from one species can help to focus efforts on antigen screening in the other species.

#### 4.1.3 Vaccination with schizont antigens

Five of the *T. parva* antigens recognized by CD8 T cells, including Tp1 and Tp2, have also been tested for their ability to induce immune responses and protection against parasite challenge.^71^ These experiments involved the use of prime–boost protocols involving priming with plasmid DNA or recombinant canarypox viruses followed by a single boost with replication‐defective recombinant vaccinia viruses (Ankara strain). Animals were immunized simultaneously with five of the antigens in separate DNA or viral constructs. Most of the immunized animals (19 of 24) exhibited readily detectable antigen‐specific CD8^+^ T‐cell IFN‐γ responses following immunization, but the CD8 T cells only exhibited detectable cytotoxic activity for parasitized cells in four of the 24 immunized animals.^71^

The animals were challenged along with unimmunized controls with a lethal dose of sporozoites 3 weeks after the booster immunization. Nine of the 19 immunized animals that generated a specific CD8 T‐cell IFN‐γ response, including the four whose CD8 T cells exhibited cytotoxicity, survived the challenge, although some of them developed moderate‐to‐severe clinical reactions prior to recovery. There was a significant association between survival and the induction of a cytotoxic CD8 T‐cell response. Thus, although the immunization protocols used in this study clearly did not induce a level of immunity that is of practical value for vaccination, they resulted in reduction in severity of disease and survival in a proportion of the animals.

Subsequent experiments in which similarly immunized cattle were treated with DNA‐encoded FLT3L and GM‐CSF, in an attempt to increase targeting of the expressed antigen to dendritic cells, failed to enhance protection.^76^

The results obtained from these initial vaccination experiments indicated that the vaccine‐induced CD8 T‐cell responses to schizont antigens may not be fully functionally competent to provide protection against parasite challenge. Current studies are investigating differences in the function of the specific CD8 T cells induced by live parasites and the subunit proteins and exploring the utility of alternative antigen delivery systems, including improved replication‐defective poxvirus vectors and other alternative viral vectors. The potential contribution of other cell subsets, such as CD4 T cells, to protection is also being investigated.

### 4.2 Protective immune responses to sporozoites

Although infections with *T. parva* 77 or *T. annulata* 78 induce only low levels of antibody against sporozoite antigens, antibodies capable of fully neutralizing the infectivity of sporozoites in vitro have been detected in animals subjected to repeated sporozoite challenge. Moreover, monoclonal antibodies with neutralizing activity (nmAbs) have been produced for both parasites by immunizing mice with sporozoites.^79^, ^80^, ^81^ The majority of such antibodies recognize a sporozoite surface protein, called p67 in *T. parva* 82 and SPAG1 in *T. annulata*.^81^ Allelic variants of the SPAG1 protein are detected in parasites derived from both cattle and Asian buffalo.^83^ While allelic variants of p67 have been detected, they appear to be found primarily in parasites derived from the African buffalo rather than from cattle‐derived parasites.^84^, ^85^

The p67^86^ and SPAG1^81^ proteins are encoded by single copy genes and consist of ~700 and ~900 amino acid residues, respectively. They are major components of the sporozoite surface membrane. Like the circumsporozoite protein (CSP) of *Plasmodium* sporozoites,^87^ the p67/SPAG1 proteins play a role in host cell recognition and entry, but they are not expressed in the schizont stage. Recombinant p67^88^ and SPAG1^89^, ^90^ incorporated in adjuvant induce sporozoite neutralizing activity in cattle and induce immunity to sporozoite needle challenge in a proportion of immunized cattle. In most of these experiments, the sporozoite challenge dose was titrated to provide less than an LD_100_.

A large number of cattle laboratory challenge experiments have been carried out with various different forms of recombinant p67 with different adjuvants and vectored antigen delivery systems, which has been recently reviewed.^31^ In brief, recombinant protein gave superior results to the vectored systems used and immunity to severe ECF ranged from 20% to ~70%. A range of different clinical responses to challenge was observed, from no‐reaction to mild, moderate and severe disease. It is likely that in the first of these categories of animals, no infection occurred, as these cattle were negative by PCR. Immunity induced by SPAG1 and different forms of SPAG1 has been less well studied, but as with p67 has been found to induce significant levels of immunity (~50%) to challenge.^90^ Interestingly, the SPAG1 protein has been shown to synergize with the protective efficacy of the Tams‐1 protein^91^, ^92^ and a live attenuated *T. annulata* cell line,^93^ indicating that there is merit in assessing the role of multiple parasite antigens in increasing the efficacy of candidate vaccine antigens.

Expression of recombinant p67 in a native and stable form remains a technical challenge. Mapping of linear B‐cell epitopes on p67 has revealed that the bovine immune response to recombinant full‐length p67 is primarily directed to N‐ and C‐terminal domains, which also harbour linear epitopes recognized by nmAbs.^94^ This has led to the demonstration that an easy‐to‐produce 80 amino acid peptide at the C‐terminal end of p67, called p67C, induces the same level of immunity to full‐length p67.^95^ This immunogen is now being used to further optimize protective immune responses to ECF. A 108 amino acid C‐terminal peptide of SPAG1 fused to hepatitis B core antigen has also been shown to induce immunity equivalent to that of full‐length SPAG1.^89^

The polymorphic immunodominant molecule (PIM) in *T. parva* is also a target of murine nmAbs,^96^, ^97^ but as cattle do not make neutralizing antibodies to full‐length recombinant PIM, this antigen has not been tested in challenge experiments.^98^, ^99^ PIM is expressed in both sporozoites and schizonts. An orthologue of this protein, called TaSp 100 and TlSP,^101^ is present in *T. annulata* and *T. lestoquardi*. The PIM antigen is rich in glutamine and proline amino acid residues and consists of a complex central domain that is variable in length flanked by conserved N‐ and C‐terminal sequences.^98^ Sequence variation in the central domain results in the PIM antigen ranging in size from 62 to 112 kDa. In contrast, the TaSP antigen does not exhibit the extreme size polymorphism of PIM and is about 36 kDa in size.^100^ Although allelic variants are found, the TaSP variants do not contain the complex central domain observed in the PIM antigen. As its name implies, cattle mount a strong antibody response to PIM and this antigen is used as a serological diagnostic test as a marker of *T. parva* infection.^102^ The TaSP protein has also been used to develop a serological diagnostic test as a marker of *T. annulata* infection.^103^

## 5 Comparative *Theileria parva* and *Theileria annulata* genomics

Available information on the antigens recognized by protective immune responses in the two *Theileria* species indicates that the immune responses are directed to the products of orthologous genes and in some instances have provided evidence of antigenic cross‐reactivity. Herein, we will explore this further to consider whether there is scope for inducing protective responses that are active against both parasite species.

### 5.1 Conservation in genome architecture and gene content

The genome architecture and protein‐coding capacity of *T. parva* and *T. annulata* are remarkably similar. *Theileria parva* 104 and *T. annulata* 105 each encode four Mbp‐sized nuclear chromosomes, a small linear mitochondrial (~7 kbp) 106, 107 and circular apicoplast (~ 39 kbp) genome with a total genome size of ~8.35 Mbp. Gene density is very high in both organisms, with *T. parva* predicted to code for a larger number of chromosomal encoded proteins (4035) than *T. annulata* (3792). It should be highlighted that these data were generated over a decade ago and there has been much improvement in bioinformatics and sequencing tools in the interim. The reference *T. parva* genome has been resequenced and re‐annotated and gene models were refined using RNAseq data from the schizont stage (cited in^108^), which has resulted in substantial change in exon–intron boundaries and in the discovery of additional protein‐coding genes. A similar exercise is being undertaken for *T. annulata* (A. Pain, personal communication). Comparative data on the physical map of chromosomes suggest that the total size of the *T. parva* genome can vary between different parasite isolates.^109^ The impact of genome size variations on parasite gene content and biology, however, remains to be fully documented. Such data are beginning to accumulate for *T. parva* as more strains are sequenced^55^, ^110^, ^111^ and their impact on genotypic diversity has been briefly reviewed elsewhere.^31^

Both genomes exhibit a highly compact structure. In brief, chromosomal DNA does not contain highly repetitive DNA, and telomeres are short, and noncoding subtelomeric sequences in *T. parva* are simpler in sequence than those found in *T. annulata*.^105^ A high proportion of protein‐coding genes encode introns that tend to be short, and intergenic regions are also short. The *T. parva* and *T. annulata* genomes exhibit near complete synteny across all four chromosomes, with interruptions due to insertions or deletions of members of the multigene families.^105^, ^112^ The most notable inversion point relates to the *T. parva* Tpr locus, which occupies a region of ~150 kbp within chromosome 3 and consists of a large tandem array of open reading frames. Members of the equivalent gene family in *T. annulata*, Tar, are dispersed across the nuclear genome. There are no interchromosomal rearrangements.

### 5.2 Conservation of candidate *Theileria parva* and *Theileria annulata* vaccine antigens

#### 5.2.1 Sporozoite antigens

Although the p67 and SPAG1 proteins exhibit only 47% sequence identity, there is sufficient conservation of epitopes between them so that anti‐p67 serum recognizes SPAG1 in immunoblots and neutralizes in vitro the infectivity of *T. annulata* sporozoites, and *vice versa*.^113^ This functionality even extends to some nmAbs. mAb 23F raised to p67 inhibits *T. annulata* sporozoite infectivity, and mAb 1A7 raised to SPAG1 inhibits *T. parva* sporozoite infectivity. By Pepscan analysis on p67, 1A7 has been shown to bind to the core peptide sequence PSLVITD. The sequence PSLVI is present in SPAG1.^113^ mAb 23F binds a conformational epitope in p67, which remains to be mapped. An orthologue of p67/SPAG1 is also present in *T. lestoquardi*, called SLAG1.^114^ Encoding 723 amino acid residues, the protein shares 42% and 58% sequence identity with p67 and SPAG1, respectively. The higher level of sequence identity with SPAG1 is perhaps not unexpected as *T. lestoquardi* is a closer relative to *T. annulata* than *T. parva* (reviewed in Ref. 115) Abs to a fragment of SLAG1 bind to p67 and SPAG1 and SLAG1 also contains the sequence PSLVI, which predicts that 1A7 should bind and inhibit *T. lestoquardi* sporozoites and that these three molecules are antigenically related.^115^ The ability of p67 and SPAG1 to induce cross‐species immunity has been confirmed,^116^ namely that p67 immunized cattle can exhibit immunity to *T. annulata* sporozoite challenge and *vice versa*. Both antigens induced approximately 50% immunity to the homologous and heterologous sporozoite challenge. The role of SLAG1 as a candidate vaccine antigen remains to be tested, but given that p67 also protects a proportion of cattle against ECF under field conditions, that is, through parasite tick challenge,^117^ this group of related proteins are prime candidate vaccine antigens.

#### 5.2.2 Schizont antigens

As discussed above, CD8 T‐cell responses specific for *T. annulata* have been shown to recognize orthologues of two of the *T. parva* CD8 T‐cell target antigens identified to date. Moreover, the amino acid sequence of the epitope identified in one of these antigens (Ta5) 74 was identical to that in the Tp5 protein,^72^ although it was presented by a different, but related MHC class I allele (1*00902 presented Tp5 and 1*02301 presented Ta5). Comparison of the predicted amino acid sequences of the 10 identified *T. parva* CD8 antigens with their *T. annulata* orthologues, based on the respective reference genome sequences, reveals a high level of sequence conservation between the orthologues of a number of the antigens (Table 1). Thus, Tp4, Tp5, Tp6 and Tp7 exhibit >96% sequence identity and Tp8 and Tp10 between 89% and 92% identity with their *T. annulata* counterparts. Remarkably, the sequences of the CD8 T‐cell epitopes identified in four of these antigens are also completely conserved between the species. The lower levels of identity observed for Tp1, Tp2 and Tp9 are consistent with previous evidence that these antigens are highly polymorphic in *T. parva* and, in the case of Tp9, also *T. annulata*.

The question of whether or not there is any cross‐protection between these Theileria species has not been addressed in any recent studies although a review by Neitz in 1957^4^ reported that animals recovered from infection with *T. annulata* remained susceptible to *T. parva*. However, in *T. parva,* CD8 T‐cell responses are often dominated by T cells specific for more polymorphic antigens^30^; hence, any cross‐immunity might only be partial and/or only apparent in a subset of animals. Evolutionary divergence of pathogen species within the same host is usually associated with antigenic divergence to minimize interspecies competition. However, as the geographical distributions of *T. parva* and *T. annulata* do not overlap, due to the different distribution of their tick vectors, there may have been minimal selective pressure to maintain antigenic divergence between these species. In the absence of a proven antigen delivery system for induction of protective CD8 T‐cell responses with defined antigens, it is currently not possible to determine the protective potential of these conserved antigens. In the meantime, further studies to examine potential cross‐protection between the two species, linked to analyses of the specificity of the T‐cell responses, may help to shed light on this question.

## 6 Conclusions

As described above, there is a great deal of similarity in the immunopathology, genomics and biology of *T. parva* and *T. annulata*. Similar protective immune responses are directed against the sporozoite and schizont stages of the parasites, and it is remarkable that many candidate sporozoite and schizont antigens are also so similar to each other. Hence, advances in development of subunit vaccines against one parasite species are likely to be readily applicable to the other. The data on antigenic conservation between the two species are contrary to expectation, as early research reported that cattle immunized with live parasites exhibited species‐specific immunity^4^, suggesting that unique antigens were likely to play a role in immunity to infection. However, what we now know about the nature of the immune response to *T. parva* and *T. annulata* may offer an explanation for the apparent lack of cross‐protection. First, the sporozoite antigens are not highly immunogenic molecules per se. Although there is clear evidence that immunity can be induced experimentally with p67/SPAG1, a single infection event induces very little antibody specific for p67/SPAG1 and it is not clear whether such responses play a role in immunity in animals residing in endemic areas. Hence, this immunity could be described as “unnatural”. Second, there is marked skewing and dominance in the antigenic specificity of CD8 T‐cell responses of individual animals, which is influenced by the MHC genotype of the animal, such that strain‐specific immunity may be engendered by T‐cell responses to one or two dominant epitopes in polymorphic antigens. Therefore, there is a need to re‐examine the cross‐protective potential of *T. parva* and *T. annulata* in cattle of MHC types known to respond to conserved epitopes. Great strides have been made in unravelling candidate *Theileria* vaccine antigens, and clear progress has been made in developing species‐specific vaccines. The shared antigens raise the intriguing possibility of developing multivalent vaccines effective against the major pathogenic bovine and ovine species of *Theileria*.

## References

[1] Uilenberg G . *Theilerial* species of domestic livestock In: Irvin AD, Cunningham MP, Young AS, eds. Advances in the Control of Theileriosis. The Hague: Martinus Nijhoff Publishers; 1981:4–37.

[2] Bishop R , Musoke A , Morzaria S , Gardner M , Nene V . *Theileria*: intracellular protozoan parasites of wild and domestic ruminants transmitted by ixodid ticks. Parasitology. 2004;129:S271–S283. 15938515 10.1017/s0031182003004748

[3] Sivakumar T , Hayashida K , Sugimoto C , Yokoyama N . Evolution and genetic diversity of *Theileria* . Infect Genet Evol. 2014;27:250–263. 25102031 10.1016/j.meegid.2014.07.013

[4] Neitz WO . Theileriosis, gonderiosis and cytauxzoonosis: a review. Onderstepoort J Vet Res. 1957;27:275–430.

[5] Pipano E . Vaccination against *Theileria annulata* theileriosis In: Wright IG, ed. Veterinary Protozoan and Hemoparasite Vaccines. Boca Raton, FL: CRC Press; 1989:203–234.

[6] Irvin AD , Morrison WI . Immunopathology, immunology and immunoprophylaxis of *Theileria* infections In: Soulsby EJL, ed. Immune Responses in Parasitic Infections: Immunology, Immunopathology, and Immunoprophylaxis. Vol. III: Protozoa. Boca Raton, FL: CRC Press; 1987:223–274.

[7] Uilenberg G . General review of tick‐borne diseases in sheep and goats worldwide. Parasitologia. 1997;39:161–165. 9530703

[8] Leemans I , Brown D , Hooshmand‐Rad P , Kirvar E , Uggla A . Infectivity and cross‐immunity studies of *Theileria lestoquardi* and *Theileria annulata* in sheep and cattle. I. In vivo responses. Vet Parasitol. 1999;82:179–192. 10348097 10.1016/S0304-4017(99)00013-8 PMC7131390

[9] Hooshmand‐Rad P , Hawa NJ . Transmission of *Theileria hirci* in sheep by *Hyalomma anatolicum anatolicum* . Trop Anim Health Prod. 1973;5:103–109.

[10] Razmi GR , Hosseini M , Aslani MR . Identification of tick vectors of ovine theileriosis in an endemic region of Iran. Vet Parasitol. 2003;116:1–6. 14519321 10.1016/s0304-4017(03)00254-1

[11] Osman SA , Al‐Gaabary MH . Clinical, haematological and therapeutic studies on tropical theileriosis in water buffaloes (*Bubalus bubalus*) in Egypt. Vet Parasitol. 2007;147:337–340. 10.1016/j.vetpar.2007.03.012 17420101

[12] Young AS , Brown CGD , Burridge MJ , et al. The incidence of theilerial parasites in East African Buffalo (*Syncerus caffer*). Trop Med Parasitol. 1978;29:281–288. 103262

[13] Barnett SF . Connective tissue reactions in acute fatal East Coast fever (*Theileria parva*) of cattle. J Infect Dis. 1960;107:253–282. 13687137 10.1093/infdis/107.3.253

[14] Flack EJ , Ouhelli H . The epidemiology of tropical theileriosis (*Theileria annulata* infection in cattle) in an endemic area in Morocco. Vet Parasitol. 1992;44:51–65. 1441192 10.1016/0304-4017(92)90143-w

[15] Fawcett DW , Doxsey S , Stagg DA , Young AS . The entry of sporozoites of *Theileria parva* into bovine lymphocytes in vitro. Electron microscopic observations. Eur J Cell Biol. 1982;27:10–21. 6806100

[16] Dobbelaere DAE , Rottenberg S . *Theileria*‐induced leukocyte transformation. Curr Opin Microbiol. 2003;6:377–382. 12941408 10.1016/s1369-5274(03)00085-7

[17] Hulliger L , Wilde JHK , Brown CGD , Turner L . Mode of multiplication of *Theileria* in cultures of bovine lymphocytic cells. Nature. 1964;203:728–730. 14207267 10.1038/203728a0

[18] von Schubert C , Xue G , Schmuckli‐Maurer J , Woods KL , Nigg EA , Dobbelaere DA . The transforming parasite *Theileria* co‐opts host cell mitotic and central spindles to persist in continuously dividing cells. PLoS Biol. 2010;8:e1000499. 20927361 10.1371/journal.pbio.1000499 PMC2946958

[19] Woods KL , Theiler R , Mühlemann M , et al. Recruitment of EB1, a master regulator of microtubule dynamics, to the surface of the *Theileria annulata* schizont. PLoS Pathog. 2013;9:e1003346. 23675298 10.1371/journal.ppat.1003346 PMC3649978

[20] Morrison WI , Buscher G , Murray M , et al. *Theileria parva*: kinetics of infection in the lymphoid system of cattle. Exp Parasitol. 1981;52:248–260. 6791953 10.1016/0014-4894(81)90080-1

[21] Brown CG , Stagg DA , Purnell RE , Kanhai GK , Payne RC . Infection and transformation of bovine lymphoid cells in vitro by infective particles of *Theileria parva* . Nature. 1973;245:s101–s103. 10.1038/245101a0 4200607

[22] Morrison WI . The aetiology, pathogenesis and control of theileriosis in domestic animals In: Zientara S, Vervoerd D, Pastoret P‐P, eds. New Developments in the Main Vector‐Borne Diseases. *OIE Scientific and Technical Review* 2015;34:599–606. 10.20506/rst.34.2.2383 26601460

[23] Conrad PA , Kelly BG , Brown CGD . Intraerythrocyte schizogony of *Theileria annulata* . Parasitology. 1985;91:67–82. 3929214 10.1017/s0031182000056523

[24] McHardy N , Wekesa LS , Hudson AT , Randall AW . Anti‐theilerial activity of BW720C (buparvaquone): a comparison with parvaquone. Res Vet Sci. 1985;39:29–33. 3929346

[25] Mhadhbi M , Naouach A , Boumiza A , Chaabani F , Ben Abderazzak S , Dargouth MA . In vivo evidence for the resistance of *Theileria annulata* to buparvaquone. Vet Parasitol. 2010;169:241–247. 20185242 10.1016/j.vetpar.2010.01.013

[26] Sharifiyazdi H , Namazi F , Oryan A , Shahriari R , Razavi M . Point mutations in the *Theileria annulata* cytochrome b gene is associated with buparvaquone treatment failure. Vet Parasitol. 2012;187:431–435. 22305656 10.1016/j.vetpar.2012.01.016

[27] Pipano E , Tsur I . Experimental immunisation against *Theileria annulata* with a tissue culture vaccine. I. Laboratory trials. Refu Vet. 1966;23:186–194.

[28] Radley DE , Brown CGD , Cunningham MP , et al. East coast fever: 3. Chemoprophylactic immunization of cattle using oxytetracycline and a combination of *Theileria* strains. Vet Parasitol. 1975;1:51–60.

[29] Morrison WI , McKeever DJ . Current status of vaccine development against *Theileria* parasites. Parasitology. 2006;133(Suppl.):S169–S187. 17274845 10.1017/S0031182006001867

[30] Morrison WI , Connelley T , Hemmink JD , MacHugh ND . Understanding the basis of parasite strain – restricted immunity to *Theileria parva* . Annu Rev Anim Biosci. 2015;3:397–418. 25422856 10.1146/annurev-animal-022513-114152

[31] Nene V , Kiara H , Lacasta A , Pelle R , Svitek N , Steinaa L . The biology of *Theileria parva* and control of East Coast fever – current status and future trends. Ticks Tick Borne Dis. 2016;7:549–564. 26972687 10.1016/j.ttbdis.2016.02.001

[32] Baldwin CL , Black SJ , Brown WC , et al. Bovine T cells, B cells and null cells are transformed by the protozoan parasite *Theileria parva* . Infect Immun. 1988;56:462–467. 3123392 10.1128/iai.56.2.462-467.1988 PMC259305

[33] Spooner RL , Innes EA , Glass EJ , Brown CG . *Theileria annulata* and *T. parva* infect and transform different bovine mononuclear cells. Immunology. 1989;66:284–288. 2784413 PMC1385101

[34] Emery DL , MacHugh ND , Morrison WI . *Theileria parva* (Muguga) infects bovine T lymphocytes in vivo and induces co‐expression of BoT4 and BoT8. Parasite Immunol. 1988;10:379–391. 3262855 10.1111/j.1365-3024.1988.tb00228.x

[35] Morrison WI , MacHugh ND , Lalor PA . Pathogenicity of *Theileria parva* is influenced by the host cell type infected by the parasite. Infect Immun. 1996;64:557–562. 8550207 10.1128/iai.64.2.557-562.1996 PMC173801

[36] Tindih HS , Geysen D , Goddeeris BM , Awino E , Dobbelaere DA , Naessens J . A *Theileria parva* isolate of low virulence infects a subpopulation of lymphocytes. Infect Immun. 2012;80:1267–1273. 22202119 10.1128/IAI.05085-11 PMC3294644

[37] Houston EF , Taracha EL , Brackenbury L , et al. Infection of cattle with *Theileria parva* induces an early CD8 T cell response lacking appropriate effector function. Int J Parasitol. 2008;38:1693–1704. 18590735 10.1016/j.ijpara.2008.05.014

[38] Campbell JD , Howie SE , Odling KA , Glass EJ . *Theileria annulata* induces aberrant T cell activation in vitro and in vivo. Clin Exp Immunol. 1995;99:203–210. 7851012 10.1111/j.1365-2249.1995.tb05533.x PMC1534289

[39] Nichani AK , Craigmile SC , Spooner RL , Campbell JD . Diminished IL‐2 responses and alteration of CD2 expression on CD8 + T cells are associated with a lack of cytotoxic T cell responses during *Theileria annulata* infection. Clin Exp Immunol. 1999;116:316–321. 10337024 10.1046/j.1365-2249.1999.00895.x PMC1905272

[40] Nichani AK , Thorp BH , Brown CG , et al. In vivo development of *Theileria annulata*: major changes in efferent lymph following infection with sporozoites or allogeneic schizont‐infected mononuclear cells. Parasitology. 1999;118:327–333. 10340321 10.1017/s0031182098003904

[41] Gill BS , Bhattacharyulu Y , Kaur D , Singh A . Vaccination against bovine tropical theileriosis (*Theileria annulata*). Nature. 1976;264:355–356. 1004558 10.1038/264355a0

[42] Brown CGD . Application of in vitro techniques to vaccination against Theileriosis In: Irvin AD, Cunningham MP, Young AS, eds. Advances in the Control of Theileriosis. The Hague: Martinus Nijhoff Publishers; 1981:104–119.

[43] Emery DL , Morrison WI , Buscher G , Nelson RT . Generation of cell‐mediated cytotoxicity to *Theileria parva* (East Coast fever) after inoculation of cattle with parasitized lymphoblasts. J Immunol. 1982;128:195–200. 6798110

[44] Innes EA , Millar P , Brown CG , Spooner RL . The development and specificity of cytotoxic cells in cattle immunized with autologous or allogeneic *Theileria annulata*‐infected lymphoblastoid cell lines. Parasite Immunol. 1989;1989:57–68. 10.1111/j.1365-3024.1989.tb00648.x 2494634

[45] Cunningham MP , Brown CG , Burridge MJ , Purnell RE . Cryopreservation of infective particles of *Theileria parva* . Int J Parasitol. 1973;3:583–587. 4200405 10.1016/0020-7519(73)90082-9

[46] Cunningham MP , Brown CGD , Burridge MJ , et al. East Coast fever: titration in cattle of suspensions of *Theileria parva* derived from ticks. Br Vet J. 1974;130:179–187. 10.1016/s0007-1935(17)35836-0 4214585

[47] Radley DE , Brown CGD , Burridge MJ , et al. East coast fever: 1. Chemoprophylactic immunization of cattle against *Theileria parva* (Muguga) and five theilerial strains. Vet Parasitol. 1975;1:35–41.

[48] Burridge MJ , Morzaria SP , Kimber CD , Cunningham MP , Brown CGD . Duration of immunity to East Coast fever (*Theileria parva* infection) of cattle. Parasitology. 1972;64:511–515. 5039345 10.1017/s0031182000045571

[49] Morzaria SP , Irvin AD , Voigt WP , Taracha EL . Effect of timing and intensity of challenge following immunization against East Coast fever. Vet Parasitol. 1987;26:29–41. 3125664 10.1016/0304-4017(87)90074-4

[50] Di Giulio G , Lynen G , Morzaria S , Oura C , Bishop R . Live immunization against East Coast fever – current status. Trends Parasitol. 2009;25:85–92. 19135416 10.1016/j.pt.2008.11.007

[51] Snodgrass DR , Trees AJ , Bowyer WA , Bergman R , Daft J , Wall AE . East Coast fever: field challenge of cattle immunised against *Theileria parva* (Muguga). Trop Anim Health Prod. 1972;4:142–151. 4210445 10.1007/BF02359762

[52] Sitt T , Poole EJ , Ndambuki G , et al. Exposure of vaccinated and naive cattle to natural challenge from buffalo‐derived *Theileria parva* . Int J Parasitol Parasites Wildl. 2015;4:244–251. 26005635 10.1016/j.ijppaw.2015.04.006 PMC4437466

[53] Bishop RP , Hemmink JD , Morrison WI , et al. The African buffalo parasite *Theileria*. sp. (buffalo) can infect and immortalize cattle leukocytes and encodes divergent orthologues of Theileria parva antigen genes. Int J Parasitol Parasites Wildl. 2015;4:333–342. 26543804 10.1016/j.ijppaw.2015.08.006 PMC4589832

[54] Pelle R , Graham SP , Njahira MN , et al. Two *Theileria parva* CD8 T cell antigens both exhibit higher polymorphism in buffalo parasites than those maintained in cattle, but differ in pattern of sequence diversity. PLoS ONE. 2011;6:e19015. 21559495 10.1371/journal.pone.0019015 PMC3084734

[55] Norling M , Bishop R , Pelle R , et al. The genomes of three stocks comprising the most widely utilized live sporozoite *Theileria parva* vaccine exhibit very different degrees and patterns of sequence divergence. BMC Genom. 2015;16:729. 10.1186/s12864-015-1910-9 PMC4583173 26403690

[56] Hemmink JD , Weir W , MacHugh ND , et al. Limited genetic and antigenic diversity within parasite isolates used in a live vaccine against *Theileria parva* . Int J Parasitol. 2016;46:495–506; Epub ahead of publication. 27080723 10.1016/j.ijpara.2016.02.007 PMC4935670

[57] Dargouth MA , Ben Miled L , Bouattour A , Melrose TR , Brown CGD , Kilani M . A preliminary study on the attenuation of Tunisian schizont‐infected cell lines of *Theileria annulata* . Parasitol Res. 1996;82:647–655. 8875574 10.1007/s004360050179

[58] Skilton RA , Bishop RP , Katende JM , Mwaura S , Morzaria SP . The persistence of *Theileria parva* infection in cattle immunized using two stocks which differ in their ability to induce a carrier state: analysis using a novel blood spot PCR assay. Parasitology. 2002;124:265–276. 11922428 10.1017/s0031182001001196

[59] Emery DL , Eugui EM , Nelson RT , Tenywa T . Cell‐mediated immune responses to *Theileria parva* (East Coast fever) during immunization and lethal infection in cattle. Immunology. 1981;43:323–336. 6454652 PMC1555008

[60] Morrison WI , Goddeeris BM , Teale AJ , Groocock CM , Kemp SJ , Stagg DA . Cytotoxic T‐cells elicited in cattle challenged with *Theileria parva* (Muguga): evidence for restriction by class I MHC determinants and parasite strain specificity. Parasite Immunol. 1987;9:563–578. 3120135 10.1111/j.1365-3024.1987.tb00530.x

[61] McKeever DJ , Taracha ELN , Innes EL , et al. Adoptive transfer of immunity to *Theileria parva* in the CD8 + cellular fraction of responding afferent lymph. Proc Natl Acad Sci USA. 1984;91:1959–1963. 10.1073/pnas.91.5.1959 PMC43284 8127915

[62] Taracha ELN , Goddeeris BM , Morzaria SP , Morrison WI . Parasite strain specificity of memory cytotoxic T cells in individual animals correlates with cross‐protection in cattle challenged with *Theileria parva* . Infect Immun. 1995;63:1258–1262. 7890382 10.1128/iai.63.4.1258-1262.1995 PMC173144

[63] Taracha ELN , Goddeeris BM , Teale AJ , Kemp SJ , Morrison WI . Parasite strain specificity of cytotoxic T cell responses to *Theileria parva* is determined primarily by immunodominance. J Immunol. 1995;155:4854–4860. 7594488

[64] Goddeeris BM , Morrison WI , Toye PG , Bishop RL . Strain specificity of bovine *Theileria parva*‐specific cytotoxic T cells is determined by the phenotype of the restricting class I MHC. Immunology. 1990;69:38–44. 1968884 PMC1385717

[65] MacHugh ND , Connelley T , Graham SP , et al. CD8 T cell responses to *Theileria parva* are preferentially directed to a single dominant antigen: implications for parasite strain‐specific immunity. Eur J Immunol. 2009;39:1–11. 10.1002/eji.200939227 PMC3149124 19670382

[66] Baldwin CL , Goddeeris BM , Morrison WI . Bovine helper T‐cell clones specific for lymphocytes infected with *Theileria parva* (Muguga). Parasite Immunol. 1987;9:499–513. 2442694 10.1111/j.1365-3024.1987.tb00526.x

[67] Brown WC , Sugimoto C , Grab DJ . *Theileria parva*: bovine helper T‐cell clones specific for both infected lymphocytes and schizont membrane antigens. Exp Parasitol. 1989;69:234–248. 2529135 10.1016/0014-4894(89)90070-2

[68] Taracha EL , Awino E , McKeever DJ . Distinct CD4 + T cell helper requirements in *Theileria parva*‐immune and ‐naive bovine CTL precursors. J Immunol. 1997;159:4539–4545. 9379055

[69] Gonze G , Campbell JD , Nichani AK , Glass EJ , Spooner RL , Ahmed JS . Evidence for strain specificity in cytotoxic T‐lymphocyte‐mediated, major histocompatibility complex class I‐dependent killing of *Theileria annulata*‐infected cells. Parasitol Res. 1998;1998:593–595. 10.1007/s004360050455 9694379

[70] MacHugh ND , Burrells A , Morrison WI . Demonstration of strain specific CD8 T cell responses to *Theileria annulata* . Parasite Immunol. 2008;30:385–393. 18498311 10.1111/j.1365-3024.2008.01038.x PMC2592345

[71] Graham SP , Pelle R , Honda Y , et al. *Theileria parva* candidate vaccine antigens recognized by immune bovine cytotoxic T lymphocytes. Proc Natl Acad Sci USA. 2006;103:3286–3291. 16492763 10.1073/pnas.0511273103 PMC1413922

[72] Graham SP , Pelle R , Yamage M , et al. Characterization of the fine specificity of bovine CD8 T‐cell responses to defined antigens from the protozoan parasite *Theileria parva* . Infect Immun. 2008;76:685–694. 18070892 10.1128/IAI.01244-07 PMC2223472

[73] Connelley TK , MacHugh ND , Pelle R , Weir W , Morrison WI . Escape from CD8^+^ T‐cell response by natural variants of an immunodominant epitope from *Theileria parva* is predominantly due to loss of TCR recognition. J Immunol. 2011;187:5910–5920. 22058411 10.4049/jimmunol.1102009

[74] MacHugh ND , Weir W , Burrells A , et al. Extensive polymorphism and evidence of immune selection in a highly dominant antigen recognised by ovine CD8 T cells specific for *Theileria annulata‐*infected leukocytes. Infect Immun. 2011;79:2059–2069. 21300773 10.1128/IAI.01285-10 PMC3088144

[75] Goh S , Ngugi D , Lizundia R , et al. Identification of *Theileria lestoquardi* antigens recognized by CD8^+^ T cells. PLoS ONE. 2016;11:e0162571. 27611868 10.1371/journal.pone.0162571 PMC5017765

[76] Mwangi DM , Honda Y , Graham SP , et al. Treatment of cattle with DNA‐encoded Flt3L and GM‐CSF prior to immunization with *Theileria parva* candidate vaccine antigens induces CD4 and CD8 T cell IFN‐γ responses but not CTL responses. Vet Immunol Immunopathol. 2011;140:244–251. 21288576 10.1016/j.vetimm.2010.12.013

[77] Musoke AJ , Nantulya VM , Buscher G , Masake RA , Otim B . Bovine immune response to *Theileria parva*: neutralizing antibodies to sporozoites. Immunology. 1982;45:663–668. 6802745 PMC1555427

[78] Gray MA , Brown CGD . In vitro neutralisation of theilerial sporozoite infectivity with immune serum In: Irvin AD, Cunningham MP, Young AS, eds. Advances in the Control of Theileriosis. The Hague: Martinus Nijoff Publishers; 1981:127–131.

[79] Dobbelaere DA , Spooner PR , Barry WC , Irvin AD . Monoclonal antibody neutralizes the sporozoite stage of different *Theileria parva* stocks. Parasite Immunol. 1984;6:361–370. 6433306 10.1111/j.1365-3024.1984.tb00808.x

[80] Musoke AJ , Nantulya VM , Rurangirwa FR , Buscher G . Evidence for a common protective antigenic determinant on sporozoites of several *Theileria parva* strains. Immunology. 1984;52:231–238. 6203832 PMC1454614

[81] Williamson S , Tait A , Brown D , et al. *Theileria annulata* sporozoite surface antigen expressed in *Escherichia coli* elicits neutralizing antibody. Proc Natl Acad Sci USA. 1989;86:4639–4643. 2499888 10.1073/pnas.86.12.4639 PMC287326

[82] Dobbelaere DA , Shapiro SZ , Webster P . Identification of a surface antigen on *Theileria parva* sporozoites by monoclonal antibody. Proc Natl Acad Sci USA. 1985;82:1771–1775. 3920654 10.1073/pnas.82.6.1771 PMC397354

[83] Katzer F , Carrington M , Knight P , et al. Polymorphism of SPAG‐1, a candidate antigen for inclusion in a sub‐unit vaccine against *Theileria annulata* . Mol Biochem Parasitol. 1994;67:1–10. 7838169 10.1016/0166-6851(94)90090-6

[84] Nene V , Musoke A , Gobright E , Morzaria S . Conservation of the sporozoite p67 vaccine antigen in cattle‐derived *Theileria parva* stocks with different cross‐immunity profiles. Infect Immun. 1996;64:2056–2061. 8675307 10.1128/iai.64.6.2056-2061.1996 PMC174036

[85] Sibeko KP , Geysen D , Oosthuizen MC , et al. Four p67 alleles identified in South African *Theileria parva* field samples. Vet Parasitol. 2010;167:244–254. 19836893 10.1016/j.vetpar.2009.09.026

[86] Nene V , Iams KP , Gobright E , Musoke AJ . Characterisation of the gene encoding a candidate vaccine antigen of *Theileria parva* sporozoites. Mol Biochem Parasitol. 1992;51:17–27. 1565135 10.1016/0166-6851(92)90196-q

[87] Coppi A , Natarajan R , Pradel G , et al. The malaria circumsporozoite protein has two functional domains, each with distinct roles as sporozoites journey from mosquito to mammalian host. J Exp Med. 2011;208:341–356. 21262960 10.1084/jem.20101488 PMC3039851

[88] Musoke AJ , Morzaria S , Nkonge C , Jones E , Nene V . A recombinant sporozoite surface antigen of *Theileria parva* induces protection in cattle. Proc Natl Acad Sci U S A. 1992;89:514–518. 1731322 10.1073/pnas.89.2.514 PMC48269

[89] Boulter NR , Glass EJ , Knight PA , Bell‐Sakyi L , Brown CG , Hall R . *Theileria annulata* sporozoite antigen fused to hepatitis B core antigen used in a vaccination trial. Vaccine. 1995;13:1152–1160. 8578798 10.1016/0264-410x(95)00026-w

[90] Boulter N , Brown D , Wilkie G , et al. Evaluation of recombinant sporozoite antigen SPAG‐1 as a vaccine candidate against *Theileria annulata* by the use of different delivery systems. Trop Anim Health Prod. 1999;4:A71–A77. 10.1046/j.1365-3156.1999.00453.x 10540314

[91] Gharbi M , Darghouth MA , Weir W , et al. Prime‐boost immunisation against tropical theileriosis with two parasite surface antigens: evidence for protection and antigen synergy. Vaccine. 2011;29:6620–6628. 21762754 10.1016/j.vaccine.2011.06.109

[92] Boulter NR , Brown CG , Kirvar E , et al. Different vaccine strategies used to protect against *Theileria annulata* . Ann N Y Acad Sci. 1998;849:234–246. 9668470 10.1111/j.1749-6632.1998.tb11054.x

[93] Darghouth MA , Boulter NR , Gharbi M , Sassi L , Tait A , Hall R . Vaccination of calves with an attenuated cell line of *Theileria annulata* and the sporozoite antigen SPAG‐1 produces a synergistic effect. Vet Parasitol. 2006;142:54–62. 16870344 10.1016/j.vetpar.2006.06.010

[94] Nene V , Gobright E , Bishop R , Morzaria S , Musoke A . Linear peptide specificity of bovine antibody responses to p67 of *Theileria parva* and sequence diversity of sporozoite‐neutralizing epitopes: implications for a vaccine. Infect Immun. 1999;67:1261–1266. 10024569 10.1128/iai.67.3.1261-1266.1999 PMC96455

[95] Bishop R , Nene V , Staeyert J , et al. Immunity to East Coast fever in cattle induced by a polypeptide fragment of the major surface coat protein of *Theileria parva* sporozoites. Vaccine. 2003;21:1205–1212. 12559799 10.1016/s0264-410x(02)00621-7

[96] Shapiro SZ , Fujisaki K , Morzaria SP , et al. A life‐cycle stage‐specific antigen of *Theileria parva* recognized by anti‐macroschizont monoclonal antibodies. Parasitology. 1987;94(Pt 1):29–37. 3103046 10.1017/s0031182000053427

[97] Toye PG , Musoke A , Naessens J . Role of the polymorphic immunodominant molecule in entry of *Theileria parva* sporozoites into bovine lymphocytes. Infect Immun. 2014;82:1786–1792. 24549329 10.1128/IAI.01029-13 PMC3993433

[98] Toye P , Gobright E , Nyanjui J , Nene V , Bishop R . Structure and sequence variation of the genes encoding the polymorphic, immunodominant molecule (PIM), an antigen of *Theileria parva* recognized by inhibitory monoclonal antibodies. Mol Biochem Parasitol. 1995;73:165–177. 8577324 10.1016/0166-6851(95)00110-m

[99] Toye P , Nyanjui J , Goddeeris B , Musoke AJ . Identification of neutralization and diagnostic epitopes on PIM, the polymorphic immunodominant molecule of *Theileria parva* . Infect Immun. 1996;64:1832–1838. 8613398 10.1128/iai.64.5.1832-1838.1996 PMC173999

[100] Schnittger L , Katzer F , Biermann R , et al. Characterization of a polymorphic *Theileria annulata* surface protein (TaSP) closely related to PIM of *Theileria parva*: implications for use in diagnostic tests and subunit vaccines. Mol Biochem Parasitol. 2002;120:247–256. 11897130 10.1016/s0166-6851(02)00013-0

[101] Bakheit M , Scholzen T , Ahmed JS , Seitzer U . Identification of potential antigenic proteins of *Theileria lestoquardi* . Ann N Y Acad Sci. 2006;1081:463–464. 17135549 10.1196/annals.1373.065

[102] Katende J , Morzaria S , Toye P , et al. An enzyme‐linked immunosorbent assay for detection of *Theileria parva* antibodies in cattle using a recombinant polymorphic immunodominant molecule. Parasitol Res. 1998;84:408–416. 9610640 10.1007/s004360050419

[103] Bakheit MA , Schnittger L , Salih DA , et al. Application of the recombinant *Theileria annulata* surface protein in an indirect ELISA for the diagnosis of tropical theileriosis. Parasitol Res. 2004;92:299–302. 14722760 10.1007/s00436-003-1055-7

[104] Gardner MJ , Bishop R , Shah T , et al. Genome sequence of *Theileria parva*, a bovine pathogen that transforms lymphocytes. Science. 2005;309:134–137. 15994558 10.1126/science.1110439

[105] Pain A , Renauld H , Berriman M , et al. Genome of the host‐cell transforming parasite *Theileria annulata* compared with *T. parva* . Science. 2005;309:131–133. 15994557 10.1126/science.1110418

[106] Kairo A , Fairlamb AH , Gobright E , Nene V . A, 7.1 kb linear DNA molecule of *Theileria parva* has scrambled rDNA sequences and open reading frames for mitochondrially encoded proteins. EMBO J. 1994;13:898–905. 8112303 10.1002/j.1460-2075.1994.tb06333.x PMC394889

[107] Hall R , Coggins L , McKellar S , Shiels B , Tait A . Characterisation of an extrachromosomal DNA element from *Theileria annulata* . Mol Biochem Parasitol. 1990;38:253–260. 1691446 10.1016/0166-6851(90)90028-k

[108] Tretina K , Pelle R , Silva JC . Cis regulatory motifs and antisense transcriptional control in the apicomplexan *Theileria parva* . BMC Genom. 2016;17:128. 10.1186/s12864-016-2444-5 PMC4761415 26896950

[109] Morzaria SP , Spooner PR , Bishop RP , Musoke AJ , Young JR . SfiI, NotI polymorphisms in *Theileria* stocks detected by pulsed field gel electrophoresis. Mol Biochem Parasitol. 1990;40:203–211. 1972977 10.1016/0166-6851(90)90042-k

[110] Hayashida K , Abe T , Weir W , et al. Whole‐genome sequencing of *Theileria parva* strains provides insight into parasite migration and diversification in the African continent. DNA Res. 2013;20:209–220. 23404454 10.1093/dnares/dst003 PMC3686427

[111] Henson S , Bishop RP , Morzaria S , et al. High‐resolution genotyping and mapping of recombination and gene conversion in the protozoan *Theileria parva* using whole genome sequencing. BMC Genom. 2012;13:503. 10.1186/1471-2164-13-503 PMC3575351 22998600

[112] Weir W , Sunter J , Chaussepied M , et al. Highly syntenic and yet divergent: a tale of two Theilerias. Infect Genet Evol. 2009;9:453–461. 19460310 10.1016/j.meegid.2009.01.002

[113] Knight P , Musoke AJ , Gachanja JN , et al. Conservation of neutralizing determinants between the sporozoite surface antigens of *Theileria annulata* and *Theileria parva* . Exp Parasitol. 1996;82:229–241. 8631374 10.1006/expr.1996.0030

[114] Skilton RA , Musoke AJ , Nene V , et al. Molecular characterisation of a *Theileria lestoquardi* gene encoding a candidate sporozoite vaccine antigen. Mol Biochem Parasitol. 2000;107:309–314. 10779608 10.1016/s0166-6851(99)00234-0

[115] Mans BJ , Pienaar R , Latif AA . A review of *Theileria* diagnostics and epidemiology. Int J Parasitol Parasites Wildl. 2015;4:104–118. 25830110 10.1016/j.ijppaw.2014.12.006 PMC4356873

[116] Hall R , Boulter NR , Brown CG , et al. Reciprocal cross‐protection induced by sporozoite antigens SPAG‐1 from *Theileria annulata* and p67 from *Theileria parva* . Parasite Immunol. 2000;22:223–230. 10792761 10.1046/j.1365-3024.2000.00302.x

[117] Musoke A , Rowlands J , Nene V , et al. Subunit vaccine based on the p67 major surface protein of *Theileria parva* sporozoites reduces severity of infection derived from field tick challenge. Vaccine. 2005;23:3084–3095. 15811656 10.1016/j.vaccine.2004.09.039

